# Supplementary material for: Identification of photoperiod-induced specific miRNAs in the adrenal glands of Sunite sheep (Ovis aries)
Source: Front Vet Sci. 2022 Jul 22;9:888207. doi: 10.3389/fvets.2022.888207 (PMC9354845; doi:10.3389/fvets.2022.888207)
Supplement: Supplementary file 1 [file Data_Sheet_1.ZIP › Supplementary file/miRDeep2 file.zip!/miRDeep2 file/Novel_Structure/LP42A1/Novel_512.pdf]

Provisional ID	:	NC_019466.2_16335
Score total	:	0.2
Score for star read(s)	:	-1.3
Score for read counts	:	-1.8
Score for mfe	:	1.8
Score for randfold	:	1.6
Score for cons. seed	:	
Total read count	:	8
Mature read count	:	8
Loop read count	:	0
Star read count	:	0

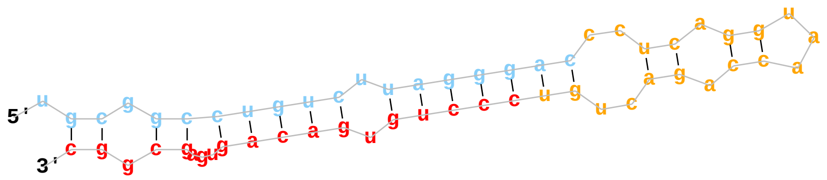

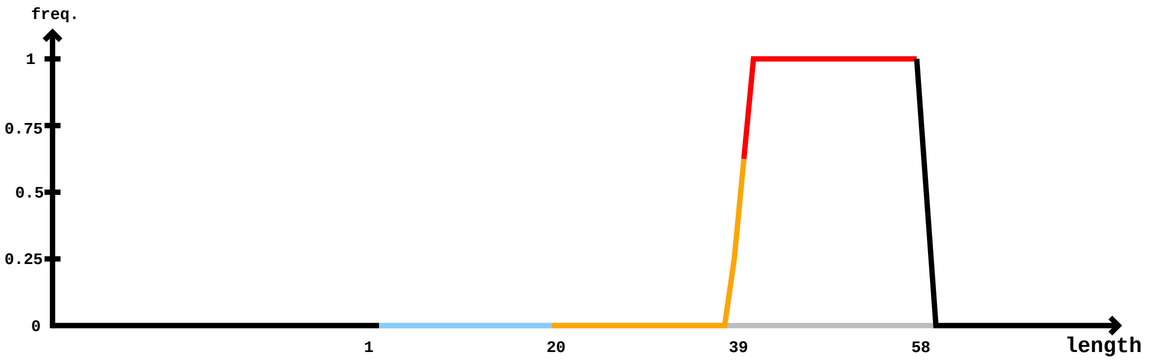


```
                                   Star                                      Mature
5'- gguuugggauaagacuuagugguuugcagaacugcggccugucuuagggacccucagguaaccagacugucccugugacagugagcggcuguaaacauccucgacugga   -3'    exp
    ((((.(((((...........((((((((..((((...(((((.(((((((..((.((...)).))..))))))).)))))...))))))))))))))))).))))...   reads    mm     sample
    .....................................................................ucccugugacagugagcggc....................   2        0      seq
    ......................................................................cccugugacagugagcggc....................   3        0      seq
    .......................................................................ccugugacagugagcggc....................   3        0      seq
```
